# Refractory eosinophilic annular erythema responsive to dupilumab: A case report and review of the literature

**DOI:** 10.1016/j.jdcr.2025.02.027

**Published:** 2025-03-12

**Authors:** Jessica Houpe, Rebecca Gibons, Molly Franklin, Austinn Miller, Andras Schaffer, Stephen K. Richardson

**Affiliations:** a Department of Dermatology, University of Central Florida/HCA Healthcare Dermatology Residency Program, Tallahassee, Florida; b Florida State University College of Medicine, Tallahassee, Florida; c Department of Dermatology, Dermatology Associates of Tallahassee, Research and Practical Scholars Program, Tallahassee, Florida

**Keywords:** case report, dupilumab, eosinophilic annular erythema

## Introduction

Eosinophilic annular erythema (EAE) is a rare figurate erythema of unknown etiology.^1^ Whether it represents a distinct clinical entity or a subset of Wells syndrome remains controversial.^1^ It most commonly presents with annular/polycyclic erythematous plaques on the trunk and/or extremities with associated tissue eosinophilia on histology. Although its etiology remains unclear, interleukin (IL) 5 has been suggested to play a role through the recruitment of eosinophils to the skin in response to an unknown allergic stimulus.^2^

We report a case of EAE presenting in an adult female with skin of color successfully treated with dupilumab. We also report our findings from descriptive analysis of all reported cases in the English medical literature to date.

## Case report

**Fig 1:**
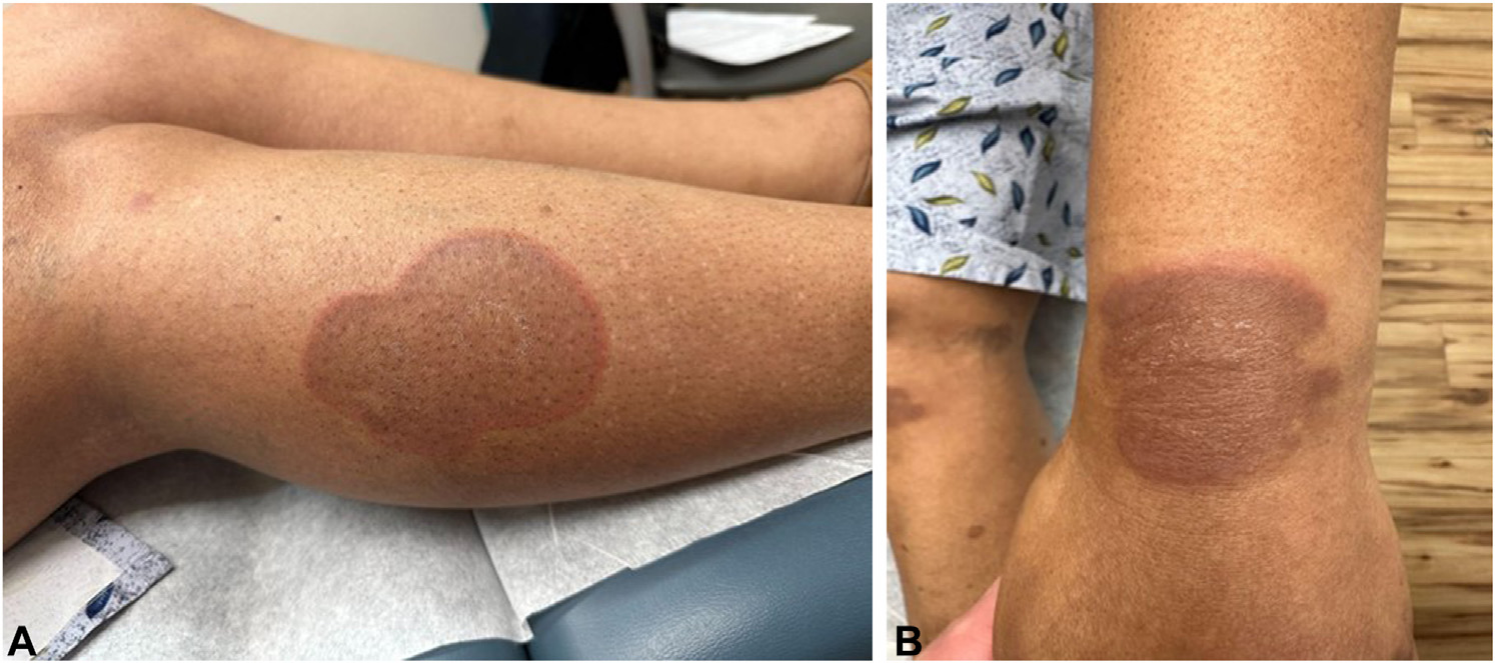
Annular *reddish-brown* plaques with elevated borders and central hyperpigmentation. **A,** Right lower leg. **B,** Left wrist.

A 65-year-old African American female with Fitzpatrick skin-type V presented with a 7-month history of a skin eruption involving her shoulders, arms, and legs. This began as small erythematous papules that centrifugally expanded over a 5-day period into large, well circumscribed, reddish-brown plaques with elevated borders (Fig 1). She denied any prodromal symptoms and exhibited a partial response to oral and topical corticosteroids.

Her past medical history was notable for bronchiectasis, Barrett’s esophagus, and *Helicobacter pylori* infection. Laboratory studies (complete blood count with differential, complete metabolic panel, thyroid stimulating hormone, urinalysis, complement levels, aldolase, Lyme serologies, strep throat culture, hepatitis panel, human immunodeficiency virus serologies, rapid plasma reagin, alpha gal antibodies, antinuclear antibodies, p- and c-antineutrophil cytoplasmic antibodies, rheumatoid factor, and serum protein electrophoresis) were unremarkable. Her chest radiograph showed stable bronchiectasis when compared to past studies.

**Fig 2:**
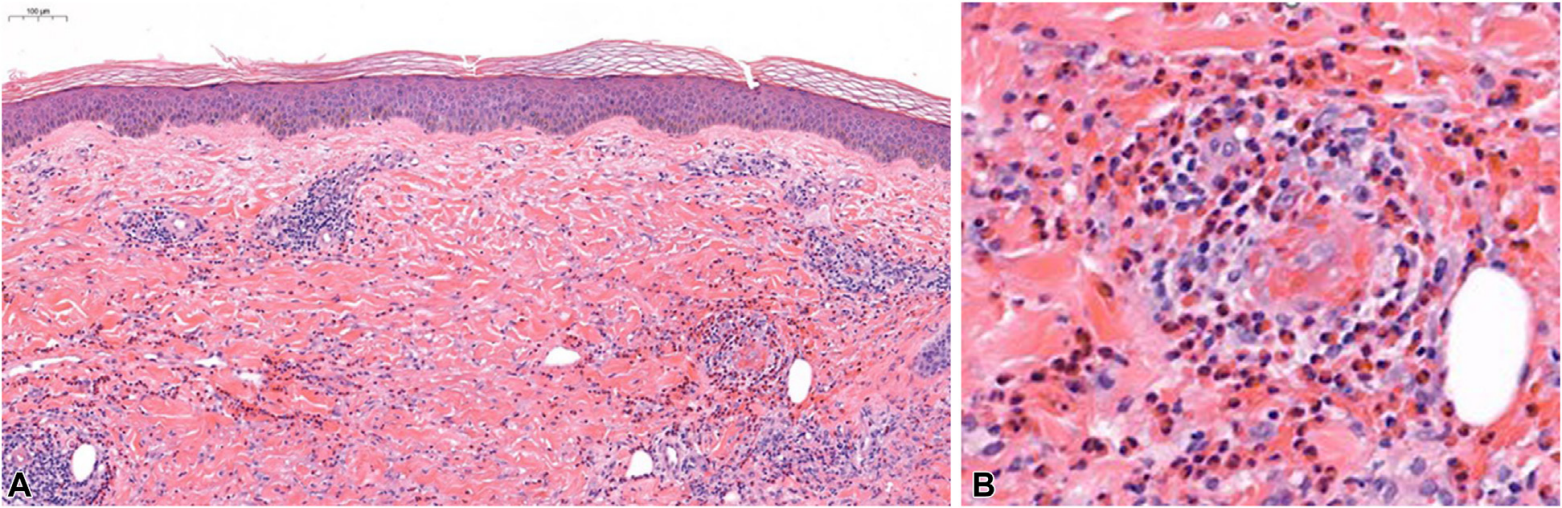
**A,** Histology showing superficial perivascular and perieccrine inflammation. **B,** Fibrinoid necrosis of vessel walls with numerous eosinophils and absence of flame figures.

A skin biopsy revealed numerous eosinophils and a lymphohistiocytic superficial perivascular and periadnexal infiltrate. Fibrinoid changes were noted within vessel walls. No flame figures were identified (Fig 2).

**Fig 3:**
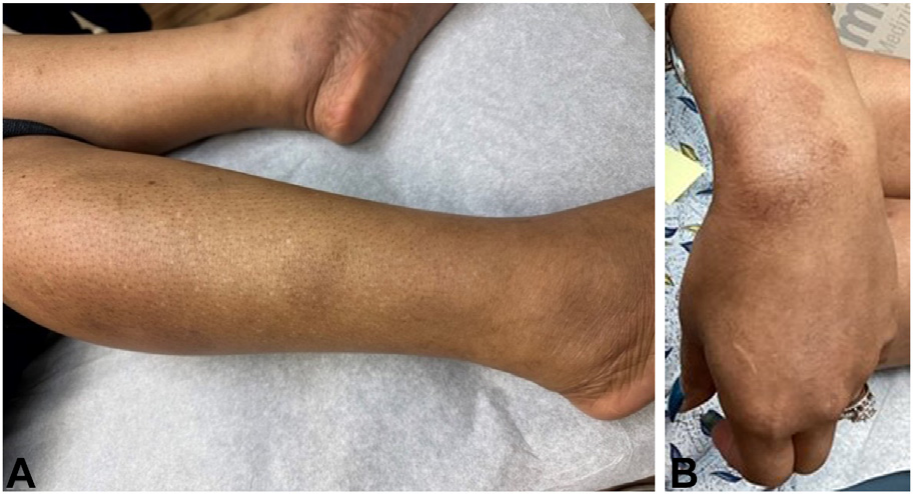
Residual postinflammatory hyperpigmentation with no evidence of active disease after 8 weeks of treatment with dupilumab. **A,** Right lower leg. **B,** Left wrist.

Based on these findings a diagnosis of EAE was established. Treatment with dupilumab was initiated (600 mg subcutaneous injection x1, followed by 300 mg subcutaneously every other week). After 8 weeks, the patient achieved complete resolution of her cutaneous disease and was subsequently transitioned to every 8-week dosing. She remains disease free 10 months to date (Fig 3).

## Discussion

EAE is a rare dermatitis that shares features with Wells syndrome. Its distinguishing characteristics include (1) the absence of prodromal symptoms (itching/burning), (2) prominent gyrate erythema, (3) the absence of “flame figures” on histology (collagen coated with eosinophil major basic protein), (4) a lack of peripheral eosinophilia, and (5) the absence of granulomas on histology.^1^ Its natural course is characterized by alternating periods of remission and relapse.^3^ The polycyclic nature of the lesions in our case appeared to represent overlapping annular plaques, a finding that is more subtle and challenging to appreciate among persons of color.

We identified 65 cases of EAE reported in the English medical literature to date. Patient data (demographics, clinical course, laboratory/imaging/pathology studies, comorbidities, treatment regimens) were compiled for descriptive statistical analysis (Supplementary Table I, available via Mendeley at https://data.mendeley.com/datasets/kg7nmzmnwm/1). Evaluation of the data revealed the following trends: (1) EAE primarily affected adult females, (2) the trunk and extremities were the most commonly affected anatomic sites, (3) associated systemic symptoms were uncommon, (4) no consistent comorbid disease associations could be identified, (5) histology primarily showed a superficial and deep perivascular and interstitial dermatitis with lymphocytes, histiocytes, and abundant eosinophils (Table I). The inconsistent histology observed in several cases was attributed to pathologic changes associated with lesion maturation.^3^ The peripheral eosinophilia reported in 14 cases was attributed to long-standing disease.^3^

**Table I:** Demographic, clinical, pathologic, comorbidity, and treatment data

Characteristic	Cases *n* (%)
Epidemiologic	
Median age (range)	52 y (3-83 y)
Female	40 (61.5%)
Race/ethnicity	
Japanese	12 (18.5%)
Middle Eastern	10 (15.4%)
White (non-Hispanic)	8 (12.3%)
African American	1 (1.5%)
Hispanic	1 (1.5%)
Not reported	33 (50.8%)
Clinical	
Duration of symptoms, median (range)	6 mo (2 d-14 y)
Location	
Trunk and/or extremities	44 (67.7%)
+face	8 (12.3%)
+genitals	1 (1.5%)
+palmoplantar (2 with face)	4 (6.2%)
Palmoplantar only	3 (4.6%)
Face only	2 (3.2%)
Neck only	1 (1.5%)
Skin folds	1 (1.5%)
Total body	1 (1.5%)
Local symptoms (*n* = 41)	
Pruritus ± burning pain	36 (55.4%)
Pain/tenderness	5 (7.7%)
None/not reported	24 (36.9%)
Systemic symptoms (*n* = 9)	
Arthralgias	2 (3.1%)
Fatigue	2 (3.1%)
Flu-like symptoms	2 (3.1%)
Cough	1 (1.5%)
Edema	1 (1.5%)
Pain	1 (1.5%)
None/not reported	56 (86.2%)
Laboratory and imaging findings (*n* = 34)	
CBC changes	16 (24.7%)
Eosinophilia	14 (87.4%)
Leukocytosis	1 (6.3%)
Neutropenia	1 (6.3%)
Infectious	4 (6.2%)
Lyme disease	3 (75%)
Blastomycosis	1 (25%)
Autoimmune antibodies	4 (6.2%)
Thyroid abnormalities	4 (6.2%)
Elevated CRP	1 (1.5%)
Elevated IgE	1 (1.5%)
Bone metastasis	1 (1.5%)
Poor metabolic control	1 (1.5%)
Polyneuropathy	1 (1.5%)
Positive urea breath test	1 (1.5%)
None/not reported	31 (47.7%)
Pathology	
Depth of inflammation	
Superficial and deep dermis	37 (56.9%)
Superficial dermis only	9 (13.8%)
Dermis, unspecified	8 (12.4%)
Superficial and mid dermis	5 (7.7%)
Epidermis with or without dermis	3 (4.6%)
Involving subcutaneous tissue	3 (4.6%)
Pattern of inflammation	
Perivascular and interstitial	32 (49.2%)
Perivascular only	13 (20%)
Interstitial only	5 (7.7%)
None/not reported	15 (23.1%)
Type of inflammation	
Eosinophils with lymphocytes with/without histiocytes with/without mast cells	53 (81.5%)
Eosinophils only	7 (10.8%)
Neutrophils with eosinophils	4 (6.2%)
Leukocytes only, no eosinophils seen	1 (1.5%)
Flame figures	
Not identified	63 (96.9%)
Identified	2 (3.1%)
Other pathologic findings	
Basal melanosis	10 (15.4%)
Vacuolar change	5 (7.7%)
Mucin	5 (7.7%)
Spongiosis	3 (4.6%)
IgM deposition	2 (3.1%)
Fibrin	1 (1.5%)
Bullae	1 (1.5%)
None/no other findings reported	38 (58.5%)
Comorbidities	
Metabolic	11 (17.0%)
Hypertension	4 (6.2%)
Diabetes	4 (6.2%)
Chronic kidney disease	3 (4.6%)
Allergy	10 (15.4%)
Asthma	9 (13.9%)
Chronic urticaria	1 (1.5%)
Infectious/Inflammatory	10 (15.4%)
Gastritis	4 (6.2%)
*H. pylori*	3 (75%)
Unstated	1 (25%)
Lyme disease	2 (3.1%)
Hepatitis C	2 (3.1%)
Appendicitis	1 (1.5%)
Tonsilitis	1 (1.5%)
Autoimmune	8 (12.4%)
Rheumatoid arthritis	4 (6.2%)
Autoimmune hepatitis/pancreatitis	2 (3.1%)
Vasculitis	2 (3.1%)
Granulomatosis with polyangiitis	1 (50%)
Eosinophilic granulomatosis with polyangiitis	1 (50%)
Malignant	5 (7.7%)
Neoplasia	4 (6.2%)
Breast	1 (25%)
Cervical	1 (25%)
Prostate	1 (25%)
Pancreatic	1 (25%)
Thymoma	1 (1.5%)
Thyroid disease	5 (7.7%)
Miscellaneous	4 (6.0%)
Mitral valve prolapse	1 (1.5%)
Psychological disease	1 (1.5%)
Uterine fibroids	1 (1.5%)
Osteoporosis	1 (1.5%)
Exposures	3 (4.6%)
Covid vaccine	2 (3.1%)
Mold exposure	1 (1.5%)
Treatment	
Systemic nonbiologic therapies	26 (40.0%)
Systemic corticosteroids	10 (15.4%)
Antimalarial	7 (10.8%)
Dapsone	4 (6.2%)
Combination of above medications	2 (3.1%)
Nicotinamide	1 (1.5%)
Suplatast tosilate	1 (1.5%)
Doxycycline	1 (1.5%)
Topical therapies	9 (13.8%)
Topical corticosteroids	8 (12.3%)
Topical immunomodulator (eg calcineurin inhibitor)	1 (1.5%)
Systemic biologic therapies	6 (9.2%)
Dupilumab	3 (4.6%)
Benralizumab	2 (3.1%)
Mepolizumab	1 (1.5%)
Spontaneous resolution	5 (7.7%)
Misc	3 (4.5%)
NB-UVB	1 (1.5%)
Thymectomy	1 (1.5%)
Discontinued culprit drug	1 (1.5%)

Reported treatments included topical therapies (corticosteroids and calcineurin inhibitors), systemic nonbiologic therapies (corticosteroids, antimalarials, dapsone, suplatast tosilate, nicotinamide, and doxycycline), systemic biologic therapies (dupilumab, benralizumab, and mepolizumab), narrow band UVB, thymectomy, and discontinuation of a suspect medication. Spontaneous resolution of disease was reported in 5 cases (Table I).

For the 3 cases treated with dupilumab, the median time to remission was 2 weeks and the median duration of remission was 20 weeks (range: 16-24 weeks). Each case was dosed at 600 mg subcutaneously on day 0, followed by 300 mg subcutaneously every 2 weeks with improvement by the second injection in all 3 cases.^4^, ^5^, ^6^ Our case achieved remission at 8 weeks and has remained disease free for 43 weeks (10 months). For the one case receiving mepolizumab, the time to remission was 4 weeks with remission lasting 24 weeks. This case was dosed at 100 mg subcutaneously every 4 weeks. The 2 cases reporting use of benralizumab did not record remission times. Both cases were dosed at 30 mg subcutaneous injection for 4 weeks for the first 3 doses, then every 8 weeks thereafter. For systemic nonbiologic therapies, the median time to remission was 2 weeks (range: 1-12 weeks) with the median remission length being 24 weeks (range: 4 weeks to 2 years).

While a wide range of topical and traditional systemic immunosuppressive/immunomodulatory therapies have been used to date with variable efficacy and inconsistent response rates, the advent of systemic biologics has offered a more targeted approach and potential for rapid and sustained clinical responses.

Dupilumab is a humanized monoclonal antibody that represents a prime molecular target for therapy.^7^, ^8^, ^9^ More specifically, dupilumab targets the IL-4 alpha receptor, thus blocking the downstream effects of IL-4 signaling (including IL-5 expression and IgE class switching). IL-5 is believed to play an essential role in EAE pathogenesis,^10^ as it promotes the growth, differentiation, recruitment, and activation of eosinophils. Our case adds to the current literature supporting the use of dupilumab for the treatment of EAE – representing the fourth published case of its use in this setting.^4^, ^5^, ^6^

Given the dearth of published reports, our analysis was limited to interpretation of general trends arising from descriptive data. As more cases are reported, we are hopeful that the validity of our observations can be tested through more rigorous inferential analysis, thus guiding future treatment recommendations for the management of this uncommon disorder.

## Conflicts of interest

None disclosed.
